# 1121. Case Study. Demonstrating the Utility of the Life Impact Burn Recovery Evaluation into Clinical Practice

**DOI:** 10.1093/jbcr/irag033.424

**Published:** 2026-04-06

**Authors:** Daniel M Schneider, Colleen M Ryan, Mary D Slavin, Jeffrey C Schneider, Huan Deng, Lewis E Kazis, Jeffrey W Shupp

**Affiliations:** The Burn Center at MedStar Washington Hospital Center, Washington, DC; Massachusetts General Hospital, Harvard Medical School, Boston, MA; Massachusetts General Hospital Brigham - Spaulding, Cohasset, MA; Spaulding Rehabilitation Hospital, Mass General Brigham, Harvard Medical School, Boston, MA; Spaulding Rehabilitation Hospital, Harvard Medical School, Boston, MA; Boston University School of Public Health, Boston, MA; MedStar Washington Hospital Center, Washington, DC

## Abstract

**Patient Presentation (age range, injury details, relevant history):**

Two adult males in their early 50s were identified to illustrate use of the Life Impact Burn Recovery Evaluation (LIBRE) in supporting behavioral health treatment, including: tracking treatment progress, targeted communication, and supporting other therapeutic interventions. Both individuals sustained burns at work and were diagnosed with posttraumatic stress disorder (PTSD) at their outpatient evaluation.

**Clinical Challenges:**

The LIBRE is a well-established and psychometrically sound assessment to measure social participation in adult burn survivors. Integration in clinical practice can enhance burn rehabilitation by offering a comprehensive, patient-centered assessment. Unfortunately, there is a disconnect between measures and their use to guide clinical practice.

**Management Approach:**

The LIBRE Profile was examined at an ABA-verified center for planning psychotherapy and monitoring progress. A licensed psychologist documented use of the LIBRE during treatment from October 2022 to September 2025. Treatment outcome measures were aimed every 4-8 weeks to assess patients’ depression (Patient Health Questionnaire-9), anxiety (Generalized Anxiety Disorder-7), posttraumatic stress (Posttraumatic Stress Disorder Checklist for DSM-5), and social participation (LIBRE).

**Outcomes:**

Patient 1 engaged in 15 outpatient psychotherapy sessions and the LIBRE was used as a self-reflective intervention promoting awareness of progress and targeted communication. Patient 2 engaged in 14 sessions and the LIBRE assisted with articulation of social reintegration challenges, self-appearance concerns, and collaboration in exposure therapy. Over the course of treatment, both individuals displayed improvements in depression, anxiety, posttraumatic stress, and social reintegration.

**Lessons Learned:**

Incorporating the LIBRE into burn rehabilitation practice enhances care quality by uniquely modifying treatment specific to burns. It aids clinicians in understanding the real-life impact of burns on survivors and facilitates a multidimensional approach to recovery. Further research on a larger scale is necessary to identify the best practices for its utilization and integration into clinical practice. We propose use of the LIBRE at the initial evaluation and prior to planned termination of services.

**Applicability to Practice:**

Use of the LIBRE in measurement-based care is considered an evidenced-based practice to support an individual’s functioning as a special type of applied psychological assessment. The LIBRE and its innovative use is a clinical intervention. The LIBRE tool also facilitates bi-directional learning and helps clinicians better understand the individual’s experience.

